# Association between Body Iron Status and Cognitive Task Performance in a Nationally Representative Sample of Older Adults

**DOI:** 10.14336/AD.2019.0064

**Published:** 2024-05-08

**Authors:** Jianying Peng, Buyun Liu, Wei Tan, Shouzhang Hu, Benchao Li, Jin Zhou, Guifeng Xu, Yangbo Sun, Linda G. Snetselaar, Robert B. Wallace, Shuang Rong, Wei Bao

**Affiliations:** ^1^Academy of Nutrition and Health, Department of Nutrition and Food Hygiene, Hubei Province Key Laboratory of Occupational Hazard Identification and Control, School of Public Health, Wuhan University of Science and Technology, Wuhan, Hubei, China.; ^2^Department of Nursing, The First Affiliated Hospital of USTC, Institute of Public Health Sciences, Division of Life Sciences and Medicine, University of Science and Technology of China, Hefei, Anhui, China.; ^3^Chief Physician/Professor, Wuhan University of Science and Technology Hospital, Wuhan, Hubei, China.; ^4^Medical Imaging Department, Wuhan University of Science and Technology Hospital, Wuhan, Hubei, China.; ^5^Shiyan Center for Disease Control and Prevention, Shiyan, China.; ^6^Department of Pediatrics, The First Affiliated Hospital of USTC, Institute of Public Health Sciences, Division of Life Sciences and Medicine, University of Science and Technology of China, Hefei, Anhui, China.; ^7^Department of Epidemiology, College of Public Health, University of Iowa, Iowa, IA 52242, USA.; ^8^Department of Endocrinology, The First Affiliated Hospital of USTC, Institute of Public Health Sciences, Division of Life Sciences and Medicine, University of Science and Technology of China, Hefei, Anhui, China.

**Keywords:** ferritin, cognitive function, aging, cross-sectional study

## Abstract

Iron is an essential micronutrient that is necessary for proper cognitive function. However, the dose-response relationship between body iron status and cognitive function remains unclear. The objective of this study was to investigate the association between serum ferritin concentrations, an indicator of body iron status, and cognitive function in older adults. Based on the National Health and Nutrition Examination Survey (NHANES) 1999-2002 in the United States, nationally representative data was collected from 2,567 adults aged 60 years and older who had objectively measured serum ferritin levels and cognitive performance. High ferritin levels were defined as a serum ferritin level >200 ng/mL in women and >300 ng/mL in men. Low ferritin levels were defined as a serum ferritin level <30 ng/mL. The digit symbol substitution test (DSST) was employed to assess cognitive function. Multivariable logistic regression analyses with survey weights were performed after the DSST was dichotomized at the median score. The weighted prevalence of adults with normal, low, and high serum ferritin levels were 73.98%, 9.12%, and 16.91%, respectively. A U-shaped association between serum ferritin concentrations and cognitive task performance was observed. After adjusting for demographic, socioeconomic, lifestyle, and C-reactive protein factors, the odds ratio (95% confidence intervals) for lower cognitive performance was 1.39 (1.11, 1.74) in adults with high ferritin levels and 1.38 (0.86, 2.22) in adults with low ferritin levels, compared with those with normal ferritin levels. The association between serum ferritin levels and lower cognitive performance was stronger in adults aged 60 to 69 years old than those aged 70 years and older. In conclusion, in a nationally representative sample of older adults in the United States, a high serum ferritin level was significantly associated with worse cognitive task performance. Thus, the relationship between low serum ferritin concentrations and cognitive task performance warrants further investigation.

## INTRODUCTION

Expanding population aging due to increased life expectancy has resulted in an increased prevalence of neurodegenerative diseases in the United States and worldwide. Accordingly, it is particularly important to identify risk factors for brain aging and cognitive performance.

Iron is an essential micronutrient and the most abundant metal in the brain. An appropriate amount of iron is necessary for proper cognitive function [1]. Iron deficiency during both development and adulthood results in diverse effects on neural function [2]. The widespread use of iron-fortified foods as well as iron supplements has decreased the prevalence of deficiency. However, iron-augmented diets may increase the risk of iron overload, especially in an iron-replete population. In recent years, the effects of “overloaded” iron status on the brain has attracted more interest [3, 4]. Iron overload, resulting in iron accumulation in the brain, has been associated with significant cognitive deficits [1, 5].

Ferritin, the major iron storage protein, has a crucial role in maintaining iron homeostasis. It was discovered that serum ferritin is an effective tool for monitoring body iron reserves [6, 7]. Moreover, serum ferritin is usually considered a more sensitive and accurate gauge of body iron stores than serum iron [8]. Ferritin can cross the blood-brain barrier [9]. House et al. found that iron levels in specific gray matter regions of older men were affected by their systemic iron status [10]. However, the relationship between serum ferritin and brain iron content is unclear, which warrants further study.

Previous studies have reported a significant association between elevated ferritin concentrations and increased risk of certain age-related chronic diseases, such as diabetes [11] and Alzheimer’s disease [12, 13]. Some studies have also researched the association between serum ferritin and cognitive performance [14-16]. However, these limited studies had inconsistent conclusions, and a larger scale study is needed to clarify the relationship between serum ferritin levels and cognition. Therefore, the present study examined the association between serum ferritin concentrations and cognitive task performance in a nationally representative sample of adults aged 60 years and older in the United States (U.S.).

## MATERIALS AND METHODS

### Study Design and Population

The National Health and Nutrition Examination Survey (NHANES), conducted by the National Center for Health Statistics at the Centers for Disease Control and Prevention, is a large-scale, multistage, ongoing, nationally representative health survey of the non-institutionalized civilian population in the U.S. The NHANES was conducted periodically before 1994, and has been a continuous program since 1999, with every two years representing one cycle consisting of approximately 10,000 participants. Details of the NHANES methods have been described extensively (www.cdc.gov/nchs/nhanes/about_nhanes.htm). The NHANES was approved by the National Center for Health Statistics Ethics Review Board. Written informed consent was obtained from all participants.

This study used data from the survey years 1999-2000 and 2001-2002, which were the only cycles that contained both serum ferritin and cognitive function measures (www.cdc.gov/nchs/nhanes/continuousnhanes/labmethods.aspx?BeginYear=1999; www.cdc.gov/nchs/nhanes/continuousnhanes/labmethods.aspx?BeginYear=2001). Cognitive task performance was assessed in participants who were aged 60 years and older. After the exclusion of participants who had missing information on serum ferritin levels and cognitive function results, 2,567 subjects remained in the final analysis.

### Measurement of Serum Ferritin Levels

Serum ferritin was measured using Bio-Rad Laboratories’ “QuantImune Ferritin IRMA” kit. In the surveys, blood was collected from study participants by venipuncture under fasting. Serum specimens were processed, stored, and shipped to the Division of Environmental Health Laboratory Sciences, National Center for Environmental Health for analysis. The laboratory methods have been described in detail elsewhere [17].

In this analysis, serum ferritin levels were classified according to previous literature [18, 19]. Normal ferritin levels were defined as a serum ferritin level higher than 30 ng/mL and lower than 200 ng/mL in women and 300 ng/mL in men. High ferritin levels were defined as a serum ferritin level higher than 200 ng/mL in women and 300 ng/mL in men. Low ferritin levels were defined as a serum ferritin level lower than 30 ng/mL [20, 21].

### Assessment of Cognitive Function

The digit symbol substitution test (DSST), a battery test of some cognitive domains, was conducted in the NHANES 1999-2002 among participants aged 60 years and older during household interviews. The DSST is a subtest of the Wechsler Adult Intelligence Scale (Third Edition), which assesses a wide array of cognitive domains, most prominently processing speed, visual-motor speed, capacity for learning, sustained attention, and working memory [22]. For the DSST, the participants copied symbols that were paired with numbers. Using the key provided at the top of the exercise form, the participants drew symbols under corresponding numbers. Sample items were provided for initial practice. The participants who were unable to complete any of the sample items did not continue with the remainder of the exercise. The number of correct symbols drawn within a period of 120 seconds was summed to yield a cognitive score, with a maximum score of 133. The more blocks correctly completed, the higher the score. To measure quality assurance, 10% of the tests were scored twice. Cognitive scores were not available for participants who refused to take the test, were unable to complete the sample items because of cognitive or physical limitations or did not complete the test within the given time limit. There is currently no consensus on how to categorize the DSST score. In this analysis, the DSST score was dichotomized at the median level. This approach has been used in previously published literature [23, 24].

### Covariate Assessment

Information on age, sex, race/ethnicity, education level, family income, smoking status, alcohol intake, and physical activity was collected using standardized questionnaires during the interviews. Race/ethnicity was classified as non-Hispanic White, non-Hispanic Black, Mexican American, or other. Education level was categorized as low (less than 9th grade, 9th to 11th grade) and high (high school graduate, some college, college graduate, and above). Family income-to-poverty ratios (IPRs) were categorized as ≤1.30, 1.31 to 3.50, and >3.50, and a higher IPR represented a higher family income status. The participants were categorized as non-smokers, past smoker, and current smoker based on their responses to questions about smoking at least 100 cigarettes during their lifetime and whether they were currently smoking. The amount of alcohol consumed was determined based on the responses to two survey queries that questioned the number of days of drinking over the past 12 months and the number of drinks per day on a given drinking day. Current alcohol intake was categorized as none (0 g/day), moderate drinker (0.1 to 27.9 g/day for men and 0.1 to 13.9 g/day for women), and heavy drinker (≥28 g/day for men and ≥14 g/day for women) [25]. For physical activity, the inactive participants were defined as those with no reported leisure-time physical activity. The active participants were defined as those who had completed recommended levels of physical activity [26] (i.e., self-reported leisure-time moderate activity [metabolic equivalents (METs) ranging from 3 to 6] of five or more times per week or leisure-time vigorous activity [METs >6] three or more times per week). The insufficiently active participants were defined as those who were not inactive but did not meet the criteria for recommended levels of physical activity. Dietary information was collected through 24-hour dietary recall interviews. Serum C-reactive protein (CRP) samples were analyzed by high-sensitivity latex-enhanced nephelometry on a BNII Nephelometer (Dade Behring). The assay used monoclonal anti-CRP antibodies and a calibrator that was traceable to the WHO Reference Material.

### Statistical Analysis

All statistical analyses accounted for the complex, multistage, stratified, and cluster-sampling design (including oversampling of certain subpopulations) of the NHANES using sample weights, strata, and primary sampling units embedded in the NHANES data. Taylor series linearization was used for variance estimation. Comparisons of the baseline characteristics of the participants with different serum ferritin levels were performed using the analysis of variance for continuous variables and the chi-squared test for categorical variables.

Multivariable logistic regression models were performed to examine the association between serum ferritin levels and the DSST scores. Model 1 was adjusted for age, gender, and race/ethnicity. Additionally, education level, family IPR, smoking status, alcohol intake, and physical activity was adjusted in Model 2. In Model 3, the inflammatory marker CRP was also adjusted because serum ferritin levels rise under inflammatory conditions [27].

To evaluate the effects of the modifications, stratified analyses were conducted according to age (60 to 69 years old and 70 to 85 years old), gender (men and women), race/ethnicity (non-Hispanic White, non-Hispanic Black, Mexican American, and other), education level (low and high) and family IPR (≤1.30, 1.31 to 3.50, and >3.50). *P* values for heterogeneity were derived from the multiplicative interaction term coefficients (exposure variable × effect modifier variable) added to the main effects multivariable model. Because serum ferritin is nonspecifically elevated in stroke or renal disease [27, 28,], which are also associated with cognitive task performance, a series of sensitivity analyses were conducted by excluding the participants who reported a history of stroke or renal disease, respectively (as shown in the Supplementary materials: Table R1-R4). Another sensitivity analysis was conducted by excluding the participants with very low cognitive scores (i.e., <5th percentile; Supplementary materials: Table R5-R6).

All statistical analyses were conducted using the survey modules in SAS software version 9.4 (SAS Institute, Cary, NC). Two-sided *p* values <0.05 were considered statistically significant.

## RESULTS

Among the 2,567 participants (mean age=70.7 years, standard error [SE]=0.3; male=49.28%) in this study, the mean serum ferritin concentration was 148.71±4.41 ng/mL. The weighted prevalence of adults with normal, low, and high ferritin levels were 73.98%, 9.12%, and 16.91%, respectively. Compared with those with normal ferritin levels, the adults with high ferritin levels were more likely to be non-Hispanic Black, physically inactive, with a low education level, and with a higher CRP level. Compared with those with normal ferritin levels, the adults with low ferritin levels were more likely to be women, non-smokers, non-drinkers, and those with a low education level (Table 1).

**Table 1:** Characteristics of the study population (n=2,567), according to serum ferritin concentrations.

Variables	Categories of Serum Ferritin Concentrations^a^		
	Low	Normal	High
**No. of participants**	234	1,899	434
**Age (years), mean (SE)**	72.36 (0.84)	70.69 (0.27)	69.86 (0.52)
**Gender, % (SE)**			
Male	35.06 (3.29)	43.45 (0.99)	47.72 (2.52)
Female	64.94 (3.29)	56.55 (0.99)	52.28 (2.52)
**Race/ethnicity, % (SE)**			
Non-Hispanic White	83.23 (4.39)	84.92 (1.74)	80.01 (2.83)
Non-Hispanic Black	4.34 (1.24)	5.79 (0.98)	9.41(1.78)
Hispanic	2.50 (0.62)	2.85 (0.64)	2.59 (0.59)
**Education, % (SE)**			
Less than high school	32.97 (4.32)	28.47 (1.32)	33.45 (3.40)
High school	33.47 (3.22)	27.69 (1.32)	32.73 (2.96)
College and above	33.56 (4.50)	43.83 (1.42)	33.82 (3.14)
**Ratio of family income to poverty, % (SE)**			
≤1.30	27.05 (4.92)	19.59 (2.44)	23.57 (3.13)
1.31-3.50	40.12 (4.25)	38.26 (1.74)	33.35 (2.65)
>3.50	24.05 (3.35)	30.84 (2.05)	29.63 (2.83)
Missing	8.79 (2.43)	11.31 (1.51)	13.44 (2.45)
**Smoking status, % (SE)**			
Non-smoker	44.81 (4.08)	48.38 (1.87)	40.83 (3.36)
Current smoker	11.88 (3.39)	11.76 (0.87)	14.80 (2.28)
Former smoker	43.31 (4.16)	39.87 (1.65)	44.37 (3.07)
**Alcohol intake^b^, % (SE)**			
Non-drinker	79.46 (3.02)	74.68 (1.97)	70.95 (2.58)
Moderate drinker	13.10 (2.74)	12.66 (1.17)	15.67 (2.03)
Heavy drinker	4.28 (1.42)	9.43 (0.91)	11.09 (1.70)
Missing	3.16 (1.18)	3.23 (0.64)	2.30 (0.73)
**Physical activity^c^, % (SE)**			
Inactive	48.38 (3.67)	49.32 (2.75)	53.36 (2.81)
Insufficient	16.09 (3.10)	15.27 (1.00)	10.40 (1.84)
Recommended level	35.53 (4.01)	35.41 (2.13)	36.24 (2.14)
**CRP (mg/dL), mean (SE)**	0.50 (0.04)	0.51 (0.02)	0.63 (0.05)
**Cognitive function scores, mean (SE)**	43.90 (1.96)	47.31 (0.64)	45.08 (1.14)
**Serum ferritin level, ng/mL, mean (SE)**	17.91 (0.60)	107.30 (1.24)	410.90 (12.08)

Abbreviations: SE: standard errors; CRP: C-reactive protein. Values are means (SE) or percentages (SE) and are weighted with respect to the number of participants.

a Normal ferritin levels were defined as a serum ferritin level higher than 30 ng/mL and lower than 200 ng/mL in women and 300 ng/mL in men. High ferritin levels were defined as a serum ferritin level higher than 200 ng/mL in women and 300 ng/mL in men. Low ferritin levels were defined as a serum ferritin level lower than 30 ng/mL.

b Non-drinker: 0 g/day; Moderate drinker: 0.1-28 g/day for men and 0.1-14 g/day for women; Heavy drinker: ≥28 g/day for men and ≥14 g/day for women.

c Insufficient activity was defined as the sum of (weekly frequency of moderate activity/5) + (weekly frequency of vigorous activity/3) <1; recommended activity was defined as the sum of (weekly frequency of moderate activity/5) + (weekly frequency of vigorous activity/3) ≥1.

The DSST scores ranged widely among the study participants, from 0 to 117 points (mean±SE: 46.59±0.62), with a median score of 42 points. The DSST scores decreased significantly with age (*p*<0.001). There was no significant difference in scores between the women and the men and with respect to tobacco use and alcohol intake. However, cognitive scores were significantly lower for the participants who had less formal education, were of different race/ethnicity groups, and had lower physical activity levels and IPRs (*p*<0.001) (Supplementary Table 1). The weighted multivariable logistic regression models examined the association between serum ferritin levels and cognitive task performance (outcome variable), as depicted in Table 2. After adjusting for demographic, socioeconomic, lifestyle, and CRP factors, the odds ratio (OR) and 95% confidence intervals (CI) for lower cognitive performance was 1.38 (0.86, 2.22) for the participants with low ferritin levels and 1.39 (1.11, 1.74) for the participants with high ferritin levels, compared with those with normal ferritin levels. Sensitivity analyses yielded results similar to those from the primary analyses (data not shown).

**Table 2:** **Association of serum ferritin concentrations with lower cognitive performance in U.S**. adults aged 60 years and older.

	Categories of Serum Ferritin Concentrations				
	Low		Normal	High	
	OR^a^ (95% CI)	*p* value	OR^a^ (95% CI)	OR^a^ (95% CI)	*p* value
**Model 1**	1.46 (0.96, 2.20)	0.07	1 (Ref)	1.46 (1.14, 1.87)	˂0.01
**Model 2**	1.38 (0.86, 2.23)	0.23	1 (Ref)	1.40 (1.12, 1.76)	˂0.01
**Model 3**	1.38 (0.86, 2.22)	0.23	1 (Ref)	1.39 (1.11, 1.74)	0.01

Model 1: Adjusted for age, gender, and race/ethnicity;

Model 2: Model 1+ education level, family income level, smoking status, alcohol consumption, and physical activity level;

Model 3: Model 2+ CRP level.

a Odds ratio for low cognitive performance, defined by the DSST score below the median (i.e., 42).

In the stratified analyses, the association between serum ferritin levels and cognitive scores was more pronounced in the participants aged 60 to 69 years old. The OR (95% CI) for lower cognitive performance in association with high ferritin levels was 1.74 (1.08, 2.80) for the participants aged 60 to 69 years old and 1.13 (0.86, 1.48) for those aged 70 years and older. The OR (95% CI) associated with low ferritin levels was 3.57 (1.65, 7.73) for the participants aged 60 to 69 years old and 0.95 (0.53, 1.69) for those aged 70 years and older. The association between high serum ferritin levels and cognitive scores was stronger in women and non-Hispanic Whites, although the effect modifications were not significant (Table 3).

**Table 3:** **Stratified analyses for the association between serum ferritin levels and lower cognitive performance in U.S**. adults aged 60 years and older.

Variables	N	Low vs Normal Ferritin Levels	High vs Normal Ferritin Levels
**Age**			
**60-69**	1,190	3.57 (1.65, 7.73)	1.74 (1.08, 2.80)
**70-85**	1,369	0.95 (0.53, 1.69)	1.13 (0.86, 1.48)
**Gender**			
**Male**	1,261	0.74 (0.43, 1.30)	1.20 (0.79, 1.83)
**Female**	1,298	1.88 (0.91, 3.90)	1.53 (1.06, 2.20)
**Race/ethnicity**			
**Non-Hispanic White**	1,545	1.27 (0.78, 2.05)	1.40 (1.05, 1.86)
**Non-Hispanic Black**	355	1.62 (0.71, 3.71)	1.80 (0.99, 3.28)
**Hispanic**	509	1.19 (0.42, 3.39)	0.50 (0.23, 1.12)
**Education level**			
**Less than high school**	1,026	1.71 (0.76, 3.82)	1.13 (0.75, 1.69)
**High school**	617	1.13 (0.59, 2.16)	1.84 (1.06, 3.19)
**College and above**	916	1.84 (0.70, 4.84)	1.15 (0.70, 1.88)
**Ratio of family income to poverty**			
**≤1.30**	635	1.91 (0.67, 5.44)	2.03 (1.02, 4.04)
**1.31-3.50**	977	1.66 (1.07, 2.60)	1.77 (1.16, 2.71)
**>3.50**	652	0.67 (0.11, 4.08)	0.55 (0.21, 1.39)
**Missing**	295	1.47 (0.32, 6.69)	1.65 (0.75, 3.66)

Adjusted for age, gender, race/ethnicity, education level, family income, smoking status, alcohol intake, physical activity, and C-reactive protein, except stratified variables.

## DISCUSSION

This large multi-ethnic/racial, nationally representative study of U.S. adults aged 60 years and older found that high serum ferritin concentrations were significantly associated with worse cognitive task performance after adjusting for established confounders. The association was stronger in the participants 60 to 69 years old than those aged 70 years and older. In addition, low serum ferritin concentrations were also associated with lower cognitive task performance, although the association was not statistically significant.

Population studies on the association between serum ferritin concentrations and cognitive function are limited [13]. A previous study reported that increased serum ferritin was associated with decreased sensorimotor speed, complex speed, and information-processing speed in older adults [15], while another study found that ferritin in women aged 35 to 60 years old was negatively correlated with phonemic fluency, composite cognitive measure, and number span/forward digit span scores [16]. A recent study of 1,030 older adults in Spain identified that lower serum ferritin levels below 39 ng/mL were associated with reduced cognitive performance in the domain of executive function [17], which was consistent with the findings in the current study. In another study, abnormal serum ferritin levels (low or high) were not associated with global cognitive performance and executive function in Australians 60 years and older; however, the discrepancy may have been due to the use of different cognitive tests (i.e., the DSST in the current study vs. the Cambridge Cognitive [CAMCOG] test) [14]. The DSST test administered in the present study was proven to be a sensitive and robust tool for detecting cognitive performance, which requires response speed, sustained attention, visual spatial skills, and set shifting [22]. Furthermore, abnormal levels of serum ferritin and brain iron have been observed in some patients with neurodegenerative diseases [29]. Elevated blood ferritin levels were also observed in patients with Alzheimer’s disease (AD) [30], and for those at the preclinical AD stage, in elderly individuals with high neocortical amyloid-β load [12]. Sternberg et al. [31] found that serum ferritin levels were significantly higher in mild cognitive impairment (MCI) and AD patients than in normal populations, and MCI patients had higher serum ferritin levels than AD patients. Abnormal accumulation of amyloid-β begins 20 years before the onset of clinical symptoms in AD patients [32]. Moreover, decreased cognitive function is often accompanied by the accumulation of amyloid-β in the brain. These results also supported the association between higher serum ferritin levels and poorer cognitive ability.

The current findings are biologically plausible. Iron is an essential micronutrient and the most abundant metal in the brain. However, as a redox-active transitional metal, excess iron accumulation can cause oxidative stress through the production of reactive oxygen species and can induce mitochondrial dysfunction and cellular damage, leading to neuronal death and functional impairment in the brain [33]. Iron overload in the brain also aggravates the production of amyloid-β, which is produced by the cleavage of APP via β secretase and γ secretase. The activity of β secretase is regulated by furin. Excessive iron concentration reduces the production of furin, increases the activity of β secretase, and then promotes the production of amyloid-β, eventually damaging cognition [34]. In addition, ApoE-e4 allele is a genetic risk factor for AD, but its mechanism is still unclear. Studies have found that iron deposition may play a role in the mechanism of increased AD risk caused by ApoE-e4 allele, and the change in ferritin content between e4 allele carriers and non-carriers has provided evidence for this hypothesis [35]. Serum ferritin is also a marker of inflammatory response, and chronic inflammation in the brain is one of the main pathological features of AD [36]. Therefore, the abnormal increases of ferritin may also be caused by the inflammatory response in early AD. This hypothesis may provide an explanation for the result that the association between serum ferritin and DSST scores was stronger in the participants aged 60 to 69 years old than those aged 70 years and older in the current study. Those aged 60 to 69 are in the early stage of cognitive decline, when the role of inflammatory reaction is more significant [37], resulting in an abnormal increase of serum ferritin. This may also serve as evidence that serum ferritin has a preferable predictive value for early AD.

There are several strengths of this study. First, the analysis was based on a multi-ethnic/racial, nationally representative sample from the NHANES, which generated findings from the general population in the U.S. Second, the use of serum ferritin data in this study provided an objective quantification of body iron status. In addition, adjusting for serum CRP accounted for potential confounding by inflammation because ferritin levels raise inflammatory status [38]. Lastly, the rich data collected by the NHANES was adjusted for a variety of potential confounders.

There are several potential limitations. First, given the cross-sectional design, the temporal relationship and causality of the association were not determined. Second, the DSST was exclusively employed in the NHANES as a neuropsychological assessment tool primarily targeting response speed, sustained attention, visual spatial skills, and set shifting. Consequently, the results from other screening instruments were not obtained. Future studies are needed to reveal the relationship between body iron status and other cognitive domains. Finally, the possibility of residual confounding by unknown or unmeasured risk factors could not be completely ruled out.

This study found that in a nationally representative population, high serum ferritin levels were significantly associated with worse cognitive performance in older adults in the U.S. More research is needed to confirm these findings and explore their underlying mechanisms.

## Supplementary Materials

The Supplementary data can be found online at: www.aginganddisease.org/EN/10.14336/AD.2019.0064.


