# Supplementary material for: Association between Body Iron Status and Cognitive Task Performance in a Nationally Representative Sample of Older Adults
Source: Aging Dis. 2024 May 8;16(2):1141–8. doi: 10.14336/AD.2019.0064 (PMC11964420; doi:10.14336/AD.2019.0064)
Supplement: Supplementary file 1 [file AD-16-2-1141-s.pdf]

SUPPLEMENTARY DATA

Association between Body Iron Status and Cognitive Task Performance in a Nationally Representative Sample of Older Adults

Jianying Peng, Buyun Liu, Wei Tan, Shouzhang Hu, Benchao Li, Jin Zhou, Guifeng Xu, Yangbo Sun, Linda G. Snetselaar, Robert B. Wallace, Shuang Rong, Wei Bao

© 2024. Peng J et al. Published online at http://www.aginganddisease.org/EN/10.14336/AD.2019.0064

SUPPLEMENTARY DATA

Supplementary Table 1. Cognitive function scores among various population groups in U.S. adults aged 60 years and older.

Variables	DSST Score Means (SE)	*p Values*
Gender		
Male	45.68 (0.78)	0.07
Female	47.29 (0.73)	
Race/ethnicity		
Non-Hispanic White	48.74 (0.79)	
Non-Hispanic Black	32.84 (1.06)	0.01
Mexican American	34.73 (1.23)	
Education level		
Low	41.32 (0.73)	<0.01
High	54.15 (0.65)	
Ratio of family income to poverty		
≤1.30	34.52 (0.89)	
1.31-3.50	45.52 (0.61)	<0.01
>3.50	55.67 (0.73)	
Missing	48.52 (1.31)	
Smoking status		
Non-smoker	46.23 (0.75)	
Current smoker	45.93 (1.21)	0.29
Past smoker	47.21 (0.92)	
Alcohol intake [a]		
Non-drinker	45.34 (0.73)	
Moderate drinker	50.09 (1.10)	0.07
Heavy drinker	53.99 (1.63)	
Missing	39.82 (2.10)	
Physical activity [b]		
Inactive	42.22 (0.68)	
Insufficient	50.25 (1.35)	<0.01
Recommended level	51.23 (0.94)	
Stroke		
Yes	36.20 (1.51)	<0.01
No	47.31 (0.62)	

Values means (SE) are weighted.

© 2024. Peng J et al. Published online at http://www.aginganddisease.org/EN/10.14336/AD.2019.0064

SUPPLEMENTARY DATA

Abbreviations: DSST: digit symbol substitution test.
[a] Non-drinker: 0 g/day; Moderate drinker: 0.1-28 g/day for men and 0.1-14 g/day for women; Heavy drinker: ≥28 g/day for men and ≥14 g/day for women.
[b] Insufficient activity was defined as the sum of (weekly frequency of moderate activity/5) + (weekly frequency of vigorous activity/3) <1; recommended activity was defined as the sum of (weekly frequency of moderate activity/5) + (weekly frequency of vigorous activity/3) ≥1.

© 2024. Peng J et al. Published online at http://www.aginganddisease.org/EN/10.14336/AD.2019.0064
